# Vulnerabilities of Venezuelan refugee women: violence and intersectional social relations^*^

**DOI:** 10.1590/1980-220X-REEUSP-2022-0430en

**Published:** 2023-10-27

**Authors:** Rubia Geovana Smaniotto Gehlen, Tassiane Ferreira Langendorf, Letícia Becker Vieira, Stela Maris de Mello Padoin

**Affiliations:** 1 Universidade Federal de Santa Maria. Programa de Pós-Graduação em Enfermagem, Santa Maria, RS, Brazil.; 2 Universidade Federal de Santa Maria. Departamento de Enfermagem. Programa de Pós-Graduação em Enfermagem, Santa Maria, RS, Brazil.; 3 Universidade Federal do Rio Grande do Sul, Escola de Enfermagem, Programa de Pós Graduação em Enfermagem, Porto Alegre, RS, Brazil.

**Keywords:** Interpersonal Relations, Health Vulnerability, Violence Against Women, Human Rights, Refugees, Relaciones Interpersonales, Vulnerabilidad en Salud, Violencia contra la Mujer, Derechos Humanos, Refugiados, relações Interpessoais, Vulnerabilidade em saúde, Violência contra a Mulher, Direitos Humanos, Refugiados

## Abstract

**Objective:**

To analyze the vulnerabilities of Venezuelan women considering their experiences of violence in refugee situations.

**Method:**

Qualitative study, developed with ten (10) Venezuelan refugee women in the southern region of Brazil, through individual in-depth interviews. The theoretical framework of analysis was Vulnerability, Human Rights, and Intersectionality.

**Results:**

The greater the intensity of the intersection of social markers present, such as sex, racial, nationality, generation, cultural, bodily, territorial and other relations, the greater the range of vulnerable experiences in the social relations of these women, producing exclusions and violation of rights.

**Conclusion:**

The situations of vulnerabilities of refugee women are enhanced as more or less social markers intersect in their life experiences and established social relationships, causing impacts that allow the transition from ‘vulnerable to violated subjects’. Thus, intersectional relationships were formed, either promoting oppression or producing resilience and resistance.

## RESUMO

**Objetivo:**

Analisar as vulnerabilidades de mulheres venezuelanas considerando suas experiências de violências na situação de refúgio.

**Método:**

Estudo qualitativo, desenvolvido com dez (10) mulheres venezuelanas refugiadas na região sul do Brasil, por meio de entrevistas individuais em profundidade. O quadro teórico de análise foi Vulnerabilidade, Direitos Humanos e Interseccionalidade.

**Resultados:**

Quanto maior a intensidade da intersecção dos marcadores sociais presentes, como as relações de gênero, raciais, de nacionalidade, geração, culturais, corporais, territoriais e outras, maior a amplitude das experiências vulnerabilizantes nas relações sociais dessas mulheres, produzindo exclusões e violação de direitos.

**Conclusão:**

As situações de vulnerabilidades das mulheres que se refugiam acentuam-se à medida que mais ou menos marcadores sociais se interseccionam nas suas experiências de vida e nas relações sociais estabelecidas, provocando impactos que viabilizam a transição de ‘sujeitos vulneráveis para vulnerados’. Conformaram-se assim, relações interseccionais que ora promoveram opressão, ora produziram resiliência e resistência.

## RESUMEN

**Objetivo:**

Analizar las vulnerabilidades de las mujeres venezolanas considerando sus experiencias de violencia en la situación de refugiados.

**Método:**

Estudio cualitativo, desarrollado con diez (10) mujeres venezolanas refugiadas en la región sur de Brasil, a través de entrevistas individuales en profundidad. El marco teórico de análisis fue Vulnerabilidad, Derechos Humanos e Interseccionalidad.

**Resultados:**

Cuanto mayor es la intensidad de la intersección de marcadores sociales presentes, como género, raza, nacionalidad, generación, relaciones culturales, corporales, territoriales y de otro tipo, mayor es el rango de experiencias vulnerabilizantes en las relaciones sociales de estas mujeres, produciendo exclusiones y violaciones de derechos.

**Conclusión:**

Las situaciones de vulnerabilidad de las mujeres que se refugian se acentúan a medida que marcadores más o menos sociales se cruzan en sus experiencias de vida y relaciones sociales establecidas, provocando impactos que posibilitan la transición de ‘sujetos vulnerables a vulnerabilizados’. Así, se formaron relaciones interseccionales que a veces promovían la opresión y a veces producían resiliencia y resistencia.

## INTRODUCTION

The common sense conception^(1)^ that diseases or socio-sanitary problems are not related to sex, color, social class, or nationality contributes to the social understanding that all people have the same chances of getting sick or of remaining healthy. In addition, the chance of recovering from a health condition or having some quality of life would be equal, regardless of their country of origin or regions where they live, the social markers of differences involved, and their living conditions^(2)^.

However, scholars point out that populations that do not have their rights respected and guaranteed present with worse health profiles, suffering, illness, and death^(3-4)^. Studies with social groups in refugee situations^(5-11)^ state that these people are on the margins of society and public health policies, as well as being potentially at greater risk for illness^(6)^. When considering the refugee situation, the population of women is more vulnerable to violence and violations of their rights^(12)^. This vulnerability is unevenly distributed within sexes, territories, and segments of society, variably at the intersection of social markers of differences^(13)^, which are defined to delimit, classify, hierarchize, and standardize sociability relationships and build attitudes of discrimination and stigmatization, enhancing inequalities^(14)^.

Thus, manifestly, the different situations of vulnerability are produced insofar as differences among people and groups, in the different social, political, cultural and economic scenarios become inequalities of rights and citizenship. By means of this, the understanding of social relations and the interference of multiple categories of differentiation (social markers) shed light on the comprehension of the unequal positioning of refugee women in society, producing different situations that can lead to illness, especially when they present life experiences permeated by violence.

Therefore, our objective is to analyze the vulnerabilities of Venezuelan women considering their experiences of violence in refugee situations. To this end, the Vulnerability and Human Rights Framework (V&HR)^(4)^ was the option for theoretical analysis, since its guiding focus is social relations, and it considers the broader context of health, understood as the health-disease process and its relationship with Human Rights and Citizenship. It acts as a point of reference for the analysis of the problem being investigated, as it identifies potential situations of vulnerability in the individual, social, and programmatic dimensions^(4)^. The results of the study will also be discussed from theoretical and conceptual positions of intersectionality^(13,15)^, which, accordingly, points out that the social problems that mainly affect women are related to the social markers of differences intersecting their lives, since women have multiple social identities according to the contexts and territories they travel through^(13)^.

## METHOD

### Design of Study

Study with a qualitative approach guided by the theoretical framework of the Vulnerability and Human Rights Framework (V&HR).

### Local

The study scenario was the municipality of Chapada, in the inland area of Rio Grande do Sul, a pioneer in volunteering for the Interiorization Program of the Ministry of Social Development and the United Nations for the reception and humanitarian aid of migrants from Venezuela^(16)^. In September 2018, approximately 50 people with migratory status of *refugee,* 12 being women, were housed in a shelter located in a rural area. The migratory status of *refugee* is granted to people who are out of their country because of a well-founded fear of persecution, violence, and/or generalized violation of human rights and who are unable or unwilling to return to their country of origin. This status is assigned to the migrant when on Brazilian lands, by the National Committee for Refugees (*Conare*)^(16)^. Group members resided in the area collectively for about six months, and then left the community to settle in the urban perimeter in rented properties.

### Population and Selection Criteria

The participants were 10 Venezuelan women belonging to this group. The inclusion criterion was being aged 18 years or older. There was no refusal; however, two women moved to other cities before the data collection period. As a way of approaching, the researcher voluntarily carried out thematic workshops on health and language accessibility, which took place weekly at the shelter, for three months. This bond was positive for the development of this study.

### Data Collection

The invitation to participate in the study was individual and with prior scheduling for an interview. The participants chose to carry out the interview at home, when they were alone, and after working hours for those who were employed. This allowed the flow of the conversation with an in-depth interview with no interruptions. Data were generated from individual, in-depth interviews, with an average duration of one hour, semi-structured for the participants’ sociodemographic data, and with open questions about the migratory aspects and the experiences of violence lived.

Participants were free to choose the language (Portuguese or Spanish) they preferred to use to establish communication with the researcher. During the established dialogue, they were questioned about the difficulty in understanding the questions. When comprehension difficulties were observed, the questions were asked with slow, paused speech, and in easy-to-understand language. The participants were able to mix Portuguese and Spanish, which facilitated communication and understanding of the dialogue. The questions guiding the development of the interviews were theoretically based on the V&HR. Data collection was carried out from February to April 2019. The closure of each interview was based on the principle of theoretical saturation, when the objective was reached, and the field stage was concluded when all the women belonging to the group were heard.

### Data Analysis and Treatment

Data organization for analysis^(17)^ took place in three stages: (a) Data organization and pre-analysis: constitution of the *corpus* of the research from the transcripts of the interviews, concurrently with the field stage; listening and exhaustive reading of the interviews; and chromatic identification of text fragments to group similar ideas according to the analysis framework^(4)^; (b) Exploration of the material: record units (words, phrases, and expressions that give meaning to the speeches content) enumerated according to the groupings and thematic categories; and (c) Treatment of the obtained results and interpretation: presumption of inferences and interpretations about the results. After data exploration and analysis, the results were discussed based on the intersectional perspective^(13,15)^, since it confronts the differences and inequalities that are built in social relationships and that are the cause of illness for those who experience them.

For the analysis, the V&RH Framework was used^(4)^, which starts from the proposition that social relations are at the base of situations of vulnerability and negligence or violation of Human Rights. It considers that the individual, social, and programmatic spheres are interrelated and interdependent. The Framework is divided into groups and subsequent thematic categories according to its three dimensions: the individual one recognizes the person as a subject of rights, dynamically in their life scenes; the social one considers social relations, the organization and citizenship milestones and the cultural scenario; and the programmatic one analyzes how much and how governments respect, protect, and promote the right to health^(4)^.

### Ethical Aspects

This study was developed following the recommendations of Resolution no. 466 of the National Health Council of Brazil^(18)^ and was approved by the Research Ethics Committee in January 2019. For the full filling of the interviews, they were recorded in audio, with the prior consent of the participants through the Free Prior and Informed Consent Form. To preserve anonymity, participants were identified using an alphanumeric code (P = participant and a number).

## RESULTS

Ten women participated in the study, all declared themselves brown, aged between 18 and 45 years old, six were in a common-law marriage, and eight had children. As for the level of education, two had finished higher education and one had unfinished higher education; two had finished high school, and one had unfinished high school, and four had incomplete primary education. All stated having their own residence and stable job in their country of origin. Regarding the family, two had their children in Brazil. In the context of the municipality, the women’s monthly income ranged from one to two minimum wages per family. The women migrated from different regions of Venezuela and did not know each other before the migration process of interiorization.

The participants reported that their migratory experiences reflected the violence experienced and the social relations established in three different contexts: the context of origin, which is Venezuela (deterritorialization); cross-border migration and arrival in the extreme north of Brazil, in the cities of Pacaraima and Boa Vista in the state of Roraima; and, later, in the municipality of Chapada, in the state of Rio Grande do Sul, where they moved to inland places (reterritorialization).

Data revealed different markers, intersected by the social relationships of Venezuelan refugee women and their interactions. They were analyzed in the three dimensions of vulnerability: individual, social, and programmatic, which were inextricably related.

### Individual Dimension

In this dimension, situations of vulnerability had an impact on the health of Venezuelan women, triggered by their experiences of violence in three contexts: **deterritorialization (Venezuela), cross-border, and reterritorialization (Brazil)**. They reported worsening of mental health and more general health problems when starting the migration process. These are related to experiences lived in the three contexts that ranged from deterritorialization to reterritorialization, with implications in the individual dimension and violation of rights:

(In Venezuela) *I developed panic attacks due to everything I went through.* [...] *I had so many experiences, violence, xenophobia* [...] *I saw my family dying malnourished, without food, searching garbage because there is no food, so this is painful*. (P1)

*What is a psychological condition* [depression] *after everything I’ve lived?* (P2)

(In Brazil) *I didn’t report* [the situation of violence] *because we are immigrants, and the old man* [aggressor] *is from there, known, so we didn’t do anything*. (P5)

*I did nothing* [in relation to the violence suffered], *because no one would believe me, but in the pastor* [aggressor], *because he is local* [Chapada-Brazil] *and I am a Venezuelan woman. So I try not to think about everything I lived with him* [aggressor], *because I suffer a lot.* (P2)

With regard to personal resources, the level of knowledge of Venezuelan women was limited regarding the types of violence against women and information about the Brazilian laws that offer protection and the means to access them.

*Violence is hitting, raping. That’s what I know.* (P9)

*I think violence is mistreatment, both aggression and verbal, judging, discriminating against people for what they are.* (P4)

*I know there is Maria da Penha* [a law to protect against domestic violence], *I received a leaflet there in Roraima* [Brazil] *that explained that* [...]. *I don’t know well* [how to access the law], *I know there’s a number to call, but I don’t know how to do it*. (P5)

*I don’t know any* [Brazilian] *law*. [...] *I studied up to 6th grade. I couldn’t study much because I had to help my parents in Venezuela.* (P1)

*I know nothing of the laws in Brazil. I don’t know what to do* [in a situation of violence]. (P8)

The participants report that in the deterritorialization process (Venezuela) they were forced to separate from their families and migrate alone, unaccompanied by a male figure. This led to the breakdown of social relationships, especially with their family, as well as to generational and cultural conflicts, due to disapproval of the decision to migrate. In reterritorialization, when women migrated with their partners, intrafamily and affective-sexual relationships, which were already fragile, remained marked by inequalities, male violence and, even, by their dependence on men.

*My mom didn’t want me to go alone*. [to Brazil], *then we fought* [...] *and now we don’t talk to each other.* (P7)

*My parents didn’t forgive me for coming to Brazil, my mother doesn’t accept my decisions*. (P10)

*Everything is chaos now because my husband only drinks* [...] *so we fight a lot.* [...] *The money I earn at the factory* [Chapada-Brazil] *I have to give to my husband*. (P8)

*My husband is very jealous, I have to be careful with what I do* [in any context]. (P3)

*My husband one day at the shelter got really mad, he wanted to hit me [...] the women of social assistance* [from Chapada-Brazil] *had to come here to protect me!* (P4)

Also in the individual dimension, the support network (family, friends, and work colleagues) was fragile. Furthermore, the participants were unable to identify assistance and health institutions as support networks and, therefore, did not form bonds in these environments.

*I feel a little abandoned without my family here* [Brazil] *I can’t count on anyone.* (P8)

*Here* [Brazil] *I only have my husband, I only have him to take care of me.* (P9)

*I didn’t make friends here.* (P4)

*People say I got a good job* [Chapada-Brazil], a*nd it seems to me as something envious! As if I, being Venezuelan, should work elsewhere*. (P10)

*I don’t know who I would ask for help* [in a situation of violence], *I don’t know what I would do.* (P7)

*I don’t seek their help* [social assistance], *because what can they help us with?* (P2)

During the process of integration into the new migrant society, women report sadness and shame for the situations experienced, especially in the cross-border context, demonstrating feelings of humiliation and inferiority.

*The first three days sleeping on the floor and not eating* [Roraima-Brazil], *so people gave us lunchboxes, but that was very humiliating for me, depending, like, on someone feeling sorry for helping, because we left Venezuela and we continue to suffer from hunger and fear here.* (P3)

*It’s very embarrassing, leaving your house and going through all that, depending on the goodwill of others, and I was used to working and having my things..* (P7)

*In my country I worked, I had my home, I lived with dignity, and there when we arrived* [Roraima-Brazil], *we were treated like garbage, like nobody. We were invisible to many people.* (P9)

*We saw everything we built in Venezuela with our work going away very quickly* [...] *it was very sad.* (P4)

However, the women demonstrated agency and resilience in the face of the adversities encountered in the migration process. Despite the correlational forces for subordination and social oppression, the mechanisms of resistance also came from intersubjective relationships in society.

*I didn’t come to Brazil to fail.* (P3)

*We arrived in Boa Vista* [Brazil] *with much hope and faith in God that everything would work out*. (P1)

*I fought for myself in Roraima* [Brazil]. *I cried at night, but during the day I worked hard.* (P2)

*All that I experienced with my husband was like a lesson for my life.* (P10)

### Social Dimension

In this dimension, the interaction contexts are evidenced as spaces of concrete experience of the intersubjectivity of Venezuelan women in the new migrant society, traversed by norms and social powers based on political, structural, economic, and cultural organizations and religious beliefs, as well as on markers of differences such as sex, race, generations, among others. In the search for access to employment and insertion in society and the labor market, women were vulnerable to suffering different forms of violence and discrimination in Brazil.

[In Roraima-Brazil] *I tried to work in family houses and clean, but nobody accepted me because I don’t speak Portuguese well.* (P1)

*I come to see work* [Chapada-Brazil] *somewhere, everyone is talking about that Venezuelan, so we feel restrained,* [...] *discriminated*. (P4)

*When I arrived in Boa Vista* [Roraima-Brazil], *a man asked if I was alone,* [...] h*e said: if you want to earn some money, meet me at Feira do Passarão, I’m there every day. You’re very pretty and you’re going to earn a lot of money, isn’t that what you came here to Brazil for?* (P7)

Racial and class relations were established as a product not only of difference, but also of social inequality in the face of identity construction in the new migrated territory. Furthermore, the established gender relations denoted power asymmetries, articulating with the categories of body, sexuality, and culture, producing violence and oppression based on gender.

*People of the same skin color are not so prejudiced against each other. But here in Chapada* [Brazil], *what I see most is white people.* (P5)

*I see that people respect people who have more money. It shouldn’t be like that, but that’s what I see here* [Brazil], *as if poor were invisible*. (P1)

*Character has nothing to do with the person’s skin color. As we Venezuelans speak another language, our character does not change because of this, but this is not what the people in this city* [Chapada-Brazil] *think, it’s what I see, what I feel.* [...] *There are men who think that as I am a single mother with three children, I have needs, of course I do* [...], bu*t they try to buy my needs, and that for me* [...] *it’s like calling me a whore! I don’t want that life either.* (P10)

*We are all equal, it’s not our nationality that changes who we are, but the only thing different is that we have a different language*. (P2)

*I have never been so scared in my life* [...] *on the crossing by bus, because I traveled 800 km* [...] *alone, and there were a lot of men watching, they were taking care of me* [...]. *And then sometimes we stopped at places, I tried not to be too far from the bus, I slept very little because I was afraid of what could happen. I didn’t even trust the man who made the crossing, he didn’t seem trustful. But these are things that we need to face to try to live a better life.* (P7)

### Programmatic Dimension

Institutionalized forms of interaction were observed, with some elements that programmatically reduced the potential conditions of vulnerability of women in the context of reterritorialization, and with the organization of local governance for access and right to health for refugee women. Four programmatic axes of analysis of the health-disease process based on Human Rights were identified in the women’s reports: availability, accessibility, quality, and acceptability of health services for the population of refugee women.

In terms of access to services, cultural interactions were established, taking the needs stemming from women’s gender and life cycles into account, with a view to improving their health status. The care offered to them proved to depend on the equitable and multidisciplinary form, with an adequate programmatic response to their needs.

[In Chapada-Brazil] *I felt very well in the care provided by the nurse, she did not treat me differently for being a migrant, and she asked me about how we took care of our health in our country, of our cultures, she was concerned about respecting that.* (P3)

*I really liked the service provided by the health professionals at the health unit* [Chapada-Brazil] *because they worried about me, because as I’m pregnant, the doctor and the nurse asked me how the Venezuelan women had their children, what we usually do. So I liked that.* (P7)

*I arrived at the center with my belly aching a lot, and the doctor called me right away for the appointment.* (P2)

*I do prenatal care with the nurse* [...] *at the health center, she assists me at the times I need.* (P7)

Two women, on the contrary, had a different perception of the care provided by health professionals to them, demonstrating that there may be heterogeneity in the cultural competences of professionals working in health services, whose technical and scientific preparation, with a cross-cultural approach, may be insufficient or inefficient.

*I realize that they* [health professionals] *don’t really know how to serve us, they don’t know our culture, our way of taking care of our children. They feel constrained.* (P10)

*I think that professionals don’t really know how to take care of us Venezuelans, and they seem to be afraid to ask, as if we’d be offended, so they tell us to do things that aren’t our habits.* (P8)

The articulation of care among assistance, prevention, and health promotion, aiming at comprehensiveness, was also evidenced and constituted an element that mitigated vulnerabilities.

*I go to the health center* [...], *I did the pap smear, and then she asked “when was your last breast exam* [mammography]*?”, and I said “I’ve never done that”, so she gave me a paper and then they called me to say when I was going to do it.* (P4)

*My son has a health problem* [...] *in the lung* [...], *so the unit helps me with everything I need, medicine, doctors, and he also spent a few days in the hospital* [hospitalized]. (P5)

*I was referred to take the maternity course* [Chapada-Brazil] *with the other women and they took me with them to see the hospital.* (P7)

On the other hand, information regarding the systematization and organization of services provided by the municipality in the network’s service flow did not reach the entire population, especially Venezuelan women, who did not obtain sufficient information about the availability of services, in addition to going through the network in a disorganized way, getting unsatisfactory responses to their health needs. This scenario increased programmatic vulnerability, essentially in situations of violence.

*I know there is a health center and a hospital, but I don’t know if there is anything else.* (P3)

*Other services, I don’t know*. (P7)

*I didn’t like the hospital* [Chapada-Brazil], *because I went there* [...] *and the doctor said “no, this is not the place for that, you have to go to the health center or the specialized doctor”,* [...] *so the doctor gave me a tranquilizer and did not examine me.* (P1) [The report describes the experience of seeking help during an anxiety crisis due to sexual abuse committed by a religious member in the context of reterritorialization in Chapada-Brazil].

**Figure 1:**
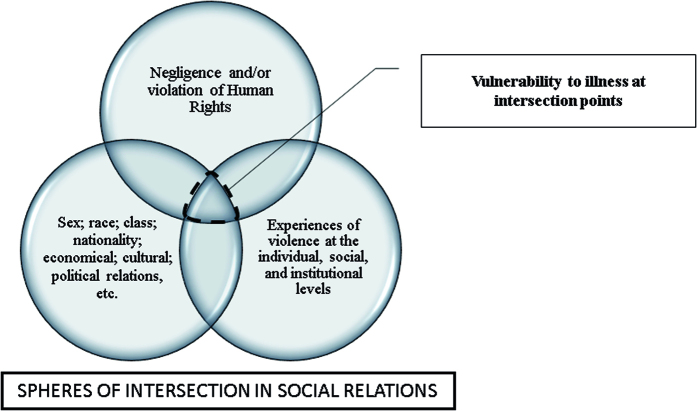
Intersection points that enhance situations of vulnerabilities.

After showing the experiences of Venezuelan refugee women in the three interrelated dimensions, we advance in the understanding that social relations, at the heart of the experiences of violence and the ways in which the latter produce situations of vulnerability, permeate the understanding that natives and refugee women cohabit under objective and subjective conditions that are the product of these relationships. On this scope, they experienced situations of vulnerabilities at the intersection points (Figure 1).

Thus, social interactions, sustained by unequal, stigmatized, and excluding intersectional relationships (of gender, race, nationality) also receive interference of violence in individual, social and institutional contexts, with consequent violation and/or neglect of rights.

## DISCUSSION

In the three contexts of migration, experiences of violence by Venezuelan women could be identified, such as domestic violence in the country of origin, gender violence at the borders, and its continuity in Brazil. Such experiences were constituted by intersectional social relations, impacted by social markers of differences, which produced exclusion and violation of Human Rights. These relationships drawn from the migratory experiences and violence of Venezuelan women led to situations of vulnerability, including mental illness, triggered according to the territories covered and the social interactions established therein, with sometimes greater predominance after the arrival on Brazilian lands, in the context of reterritorialization.

Since the refugees were unable to meet, alone, their basic needs for life and the protection of Human Rights, situations of vulnerability also permeated the context of the native country, since Venezuelan women found themselves in the aggravated scenario of social, politicial, health, and economic crisis present in the country^(16)^.

This means that the (individual, social and programmatic) construction of responses to their needs has sociability as its central determination. However, in this sociability, the starting points for refugee women in the new migrated territory tend to be exclusive due to the attribution of their differences and, consequently, due to the difficulties of emancipatory agency – of subversion of the norm – and territorial empowerment when being a foreign body^(13)^.

Thus, the intertwining of social markers of differences and their meanings in historical, political and local contexts are evident, acting as powerful analytical operators to identify the place of refugee women in society, revealing vulnerabilities. Therefore, all social relations are intersubjective, interconnected, and thus pass through the matrix of intersectionality, since they are not constituted^(13)^ by homogeneous universal beings, but by subjects with multiple identities and a plurality of facets.

The social construction of intersubjective relations is intertwined in a more complex process, which comprises the various dimensions of how society is structured and how each situation alters the composition and interweaving of class, gender, nationality, race, corporeality, culturality relations, and so many others^(19)^. However, it is important to emphasize that it is not a matter of a sum of social relations of differences, but of perceiving the new and composite reality that results from this fusion of identities and nationalities, and its real consequences, such as the intersectional violence that makes the body and mind sick.

Gender, racial, class and political identity relations as a (non) national subject do not, therefore, necessarily present themselves as a starting point of inequality and antagonism, but are found in society entangled in a network of dialectical relationships, deeply affected by the fundamental structural features of the entire social complex^(19)^. Moreover, in these tangles, intersectional violence is produced and reproduced. In terms of sociability, experiences of intersectional violence constitute a major obstacle to the full and free development of refugee women’s individuality.

The search for reterritorialization and resocialization has triggered problems for Venezuelan women since the beginning of their migration process. However, what shall be emphasized are the ways and situations in which their social markers of differences, and especially their otherness, turned into social inequalities. From them, factors of exclusion and/or discrimination caused their removal from the receiving society, provoking, in counterpoint, resistance and resilience.

Society as a whole demonstrates difficulties in naturally absorbing the presence of foreign bodies, bodies different from the Eurocentric image and female bodies outside the places culturally and historically destined for them. This potentiates the manifestation of gazes on abject bodies^(20)^ of Venezuelan refugee women, that is, “bodies that do not belong” regardless of the context in which they are inserted in their social interactions.

In this direction, society also demonstrates difficulties to understand that geographic displacement across borders configures an essential social mobility strategy for survival, being an escape from real situations of vulnerability. This situation triggers silenced intersectional vulnerabilities, allowing the construction of abyssal lines^(21)^ of gender, race, nationality, territory, and class.

The vulnerabilities crossed by the markers of difference in interactions configured articulated social relationships, that is, intersectionalized relationships. Thus, processes of stigmatization were built from the social construction in which the particular attributes of Venezuelan women disqualified them, leading to the non-full acceptance of their insertion in society, the reproduction of inequalities and discriminatory practices.

Throughout the entire migration process, even after the reterritorialization process, a double vulnerability to different forms of violence was observed. Consequently, it was found that the women became ill in view of the social reading of foreign women, that is, the participants were not seen in themselves, but through a generic image of interference of differences. Gender appears intertwined inseparably from language, class, nationality, among others.

The system of visible and invisible distinctions is made up of lines that divide social reality into two distinct universes: “on this side of the line” and “on the other side of the line”. The division is such that “the other side of the line” disappears as a reality, and becomes non-existent^(21)^. These lines, when diverse experiences in the social relations of groups that tend to be marginalized – such as refugee women – are ignored, become the production of sub-humanities and exclusions^(22)^.

With regard to moral vulnerabilities, stigmatization, racism, sexism, classism, and xenophobia were highlighted in social relations in the context of the migrant society. These are factors that imply vulnerabilities to illness, related to access to health, prevention, monitoring, and health promotion. Moreover, the processes of stigmatization maintain, for women, a constant expectation of non-acceptance, social segregation and fear of suffering more discrimination, causing psychological suffering and interfering with their quality of life and well-being^(14)^.

In the contours of human vulnerability, it is important to understand that being a refugee, by itself, does not mean being vulnerable. This is because individuals or groups are not *inherently* vulnerable, but are *temporarily* vulnerable to something, to some degree and form, at a certain point in time and space^(23)^. However, both the migration process itself and the attribution of refugee identity based on the interference of social markers, building stigmatized alterities, can favor situations of vulnerability^(24)^.

Thus, the concept of vulnerability applied to situations experienced by Venezuelan women considers them human beings in a condition of vulnerability, injured and damaged in their country of origin, requiring greater support to seek protection and guarantee of rights elsewhere^(11)^. Nevertheless, it is important to clarify that vulnerability differs from vulneration in terms of conceptual and practical meanings^(25)^.

Vulnerability refers to potential conditions of being in a situation of susceptibility to health conditions, present in human beings in general, while vulneration refers to concrete conditions, that is, it is attributed to a factual situation, causing real harm to the subject. Violations of human rights, fragility in access to health, the absence of effective public policies aimed at specific groups for health care, intersectional relationships shaping excluding identities, and the consequent situations of intersectional violence place this population, which is more vulnerable to illness, in a state of violation, leading to a significant increase in risks for the restoration of health, seen as well-being^(26)^.

Thus, Venezuelan women experienced situations of low, medium and high vulnerability, but also – and not necessarily simultaneously – found themselves vulnerable to violence and illness. In this direction, it is understood that the vulneration of Venezuelan women is the antithesis of the guarantee of their Human Rights. Ensuring human rights involves recognizing the vulnerability of these women and the need to propose the applicability of different institutional solutions for this population^(27)^.

As advances for the area of nursing and health, it is understood that, in the intersubjectivity of interactions, there is not a sum of discriminations, situations and elements that confer vulnerabilities. What exists are experiences different from those found at the intersections, and which change according to the physical and symbolic spaces and territories they travel through. There is not a closed and predetermined set of needs to be answered, as migratory contexts and interactions and social networks are constantly changing, allowing different experiences in each moment lived, and therefore demanding unique needs and arrangements in each situation. This strengthens the complexity of the analyses of this scenario, since, for its understanding, when relevant social problems are considered “universal”, either generalized explanations are sought, or one ends up producing invisibilities about their specificities.

We point out, as a limitation of the study, the cultural and language difference, which can prevent the woman from expressing herself and can distance the person who interviewed her in the data apprehension. However, to minimize this difference, the researcher, as a volunteer, carried out thematic workshops on health and linguistic accessibility before the interviews with the participants.

## CONCLUSION

The situations of vulnerabilities of refugee women are enhanced as more or less social markers intersect their life experiences and established social relationships, causing impacts that allow the transition from ‘vulnerable to violated subjects’. Thus, intersectional relationships were formed, either promoting oppression or producing resilience and resistance.

This way, social relations are also intersectional, permeated by gender, racial, nationality, generation, cultural, corporal, territorial relations, among many others. The greater the intersection of the present social markers, the greater the range of vulnerable experiences in social relationships. The use of the analytical framework showed high individual and social vulnerability for refugee women, as opposed to low programmatic vulnerability when there is a migration scenario prepared to welcome refugees.

The significance of the results of this study is also implied in the understanding that Human Rights constitute an indicator of vulnerabilities. That is, the greater the violation or neglect of these rights, the greater the situations of vulnerability. Therefore, a “Human Rights” approach intertwined with the intersectionality of social markers of women in refugee situations must be adopted by regional and local governments.

Considering the social relations of refugee women from the perspective of Human Rights vulnerability allows broadening the look at the demands of this population and direct health care, meeting specificities of each woman.
